# The Causal Effect of Maternal Education on Child Mortality: Evidence From a Quasi-Experiment in Malawi and Uganda

**DOI:** 10.1007/s13524-019-00812-3

**Published:** 2019-10-07

**Authors:** Liliana Andriano, Christiaan W. S. Monden

**Affiliations:** Department of Sociology and Nuffield College, University of Oxford, Oxford, UK

**Keywords:** Child mortality, Maternal education, Universal primary education, Instrumental variable method, Africa

## Abstract

Since the 1980s, the demographic literature has suggested that maternal schooling plays a key role in determining children’s chances of survival in low- and middle-income countries; however, few studies have successfully identified a causal relationship between maternal education and under-5 mortality. To identify such a causal effect, we exploited exogenous variation in maternal education induced by schooling reforms introducing universal primary education in the second half of the 1990s in Malawi and Uganda. Using a two-stage residual inclusion approach and combining individual-level data from Demographic and Health Surveys with district-level data on the intensity of the reform, we tested whether increased maternal schooling reduced children’s probability of dying before age 5. In Malawi, for each additional year of maternal education, children have a 10 % lower probability of dying; in Uganda, the odds of dying for children of women with one additional year of education are 16.6 % lower. We also explored which pathways might explain this effect of maternal education. The estimates suggest that financial barriers to medical care, attitudes toward modern health services, and rejection of domestic violence may play a role. Moreover, being more educated seems to confer enhanced proximity to a health facility and knowledge about the transmission of AIDS in Malawi, and wealth and improved personal illness control in Uganda.

**Electronic supplementary material:**

The online version of this article (10.1007/s13524-019-00812-3) contains supplementary material, which is available to authorized users.

## Introduction

Child survival is a key indicator of social development and remains a serious challenge for developing countries. Although child mortality has decreased dramatically since 1990, still more than 40 children per 1,000 live births died before their fifth birthday in 2016 (United Nations Inter-agency Group for Child Mortality Estimation (U.N. IGME) 2017). Most under-5 mortality occurs in sub-Saharan Africa, which has experienced a slower reduction in child mortality and currently has the highest under-5 mortality rate in the world (79 per 1,000 in 2016) (U.N. IGME 2017).

Maternal education is considered to be a key factor in reducing child mortality. Gakidou et al. (2010), for instance, estimated that increases in maternal education could account for more than 50 % of the worldwide reduction in under-5 mortality between 1970 and 2009. Many others have documented the strong negative association between maternal education and child mortality (e.g., Bicego and Boerma 1990; Caldwell 1979; Cleland et al. 1992), and this association has been a driver for big investments in the education of girls (Schultz 1993). Despite the extensive literature on maternal education and child mortality, few studies have assessed the causality of this relationship. Only four studies have used instrumental variable (IV) methods to study the nature of this relationship in just three sub-Saharan African countries: Uganda (Keats 2016; Makate 2016), Malawi (Makate and Makate 2016), and Zimbabwe (Grépin and Bharadwaj 2015). These studies show mixed results, and only three of them deal with primary education. Our analysis builds on the Universal Primary Education (UPE) reforms that were implemented in Malawi and Uganda in the 1990s. This study contributes to the existing body of work in four ways.

First, we combined individual-level survey data with official district-level statistics on the number of primary schools in order to exploit differences in district reform intensity and birth cohort as instruments for maternal education. Exposure to the UPE reforms depended on both the year and district of birth. Previous studies did not differentiate between districts in Malawi but instead controlled for four regions (Makate and Makate 2016); for Uganda, 10 large regions (Makate 2016) or an indicator for Kampala (Keats 2016) were used to control for region-specific trends. We differentiated between 27 and 38 districts that were responsible for the provision of primary schooling in Malawi and Uganda, respectively. In this way, we accounted for district-specific trends that affected both women’s educational attainment and children’s health in both countries.

Second, previous studies of Uganda and Malawi did not fully take into account censoring, thereby potentially introducing selection and recall biases. Our study provides the first IV estimates of the maternal education and under-5 mortality relationship while taking into account right-censoring. We used a Cox proportional hazard model in an IV approach, and doing so affected the results. Although linear specifications—both in this and previous studies (Keats 2016; Makate 2016)—did not provide evidence for a significant effect, we did find, using a Cox model, a significant impact of maternal education in Uganda.

Third, we added consistency to this field, with mixed designs and mixed results, by applying the same IV approach to two countries. And, finally, we added to earlier analyses by exploring a consistent set of mechanisms for the effect of maternal education in both Malawi and Uganda.

## Prior Research on the Causal Effect of Maternal Education

The negative relationship between maternal education and child mortality may be at least partly due to omitted variables, such as maternal ability, family background and resources, and community infrastructure, each of which predicts both maternal education and child mortality. Because few studies have addressed this issue empirically, we included other regions than sub-Saharan Africa in our overview.

Studies using family or household fixed effects have suggested that the effect of maternal education on child health outcomes may be overestimated. After unobserved family factors were controlled for, the association of maternal education with child outcomes was attenuated or no longer significant (see, e.g., Horton 1988; Strauss 1990; Wolfe and Behrman 1987).

Another way of addressing omitted variable bias is to use an exogenous source of variation in education that is not related to child mortality––an IV approach. IVs exploit “situations where the forces of nature or government policy have conspired to produce an environment somewhat akin to a randomized experiment” (Angrist and Krueger 2001:73). We focus on this type of quasi-experimental study. To the best of our knowledge, only six studies have examined the causal effect of maternal education on child mortality: Breierova and Duflo (2004), Chou et al. (2010), Grépin and Bharadwaj (2015), Keats (2016), Makate (2016), and Makate and Makate (2016).

Breierova and Duflo (2004) used a large-scale school construction program in Indonesia in the mid-1970s to identify the effect of parental schooling on child mortality. They measured program intensity as the number of new primary schools relative to the number of school-aged children. Using regional differences in program intensity and birth cohorts as instruments for average years of education, they found that an increase in parental education led to a significant reduction in the number of children who died and that there was no significant difference between the effect of mother’s and father’s schooling. In most cases, their two-stage least squares (2SLS) effects of parental schooling were larger than their ordinary least squares (OLS) effects.

Chou et al. (2010) exploited the 1968 expansion of compulsory schooling in Taiwan to estimate causal effects of parental educational attainment on birth outcomes. The authors instrumented parental education with the interaction between treatment status and program intensity, measured by the number of new schools relative to school-aged children. They found that maternal and paternal schooling significantly reduced the probability of neonatal, postneonatal, and infant mortality. All but one weighted 2SLS (W2SLS) effects were statistically significant, and some were larger than the weighted least squares effects.

Grépin and Bharadwaj (2015) used age-specific exposure to the 1980 expansion of access to secondary education in Zimbabwe to instrument for maternal secondary education. The expansion was part of a much wider package of reforms, introduced by the new independent government. Their IV estimates, based on the Zimbabwe Demographic and Health Surveys (DHS), indicated that maternal secondary education statistically lowered a child’s probability of dying by the time of the survey, and before ages 1 and 5. All their IV coefficients exceeded the corresponding OLS coefficients. They found that an additional year of maternal secondary schooling reduced child deaths by about 21 %. Although relevant, this study misses the majority of the population because throughout sub-Saharan Africa, especially in the 1980s, the most important and most numerous differences in education were between having no education and having a primary education.

In a recent working paper, Keats (2016) used the 1997 Ugandan UPE reform (described later) to examine the impact of female education on child health outcomes. He employed a dichotomous indicator for treatment status as instrument for maternal schooling, with women aged 7–14 in 1997 serving as the treatment group and women aged 15–22 in 1997 being the control group. His analysis, based on the Uganda DHS, was restricted to (1) women who had completed their education and (2) first births that had happened in the five years prior to the interview. The IV findings showed that an increase in women’s education affected their firstborns with regard to immunizations, preventive care, and chronic malnutrition. The IV results for infant mortality (Keats 2016:23, table 7) instead showed no evidence of significant differences between the firstborns of exposed and non-exposed women. Keats suggested that the null effect of women’s education on mortality could be because the firstborn child subsample is likely biased downward as a result of negative selection.

Makate (2016) also used the Uganda DHS and the 1997 Ugandan UPE to estimate the effect maternal education had on child mortality. He compared outcomes of children born to women aged 5–13 in 1997 (treatment group) with those of children born to women aged 17–25 in 1997 (control group). He limited the samples for infant and under-5 mortality to children who had been fully exposed to under-1 and under-5 mortality, ignoring right-censored cases still at risk of dying. This restriction introduced potential selection and recall bias. His IV estimates revealed that an additional year of maternal primary schooling negatively affected infant mortality and child death by the survey date but had no impact on under-5 mortality. The significant IV coefficients exceeded the corresponding OLS coefficients. Makate (2016) found that a one-year increase in maternal education caused a decrease of between 17.5 % and 19.3 % in child mortality; however, he warned that these results should be interpreted with caution because they were imprecise and may have lacked robustness.

Finally, Makate and Makate (2016) used the Malawian UPE reform of 1994 to examine the impact of maternal education on child mortality. Following Grépin and Bharadwaj (2015), they exploited age-specific exposure to the reform to instrument for maternal primary education, considering women aged 6–13 in 1994 as the treatment group, and women aged 17–24 in 1994 as the control group. Like Makate (2016), they too limited their samples for infant and under-5 mortality to children who had been fully exposed, thus introducing potential selection and recall bias. Using data from the Malawi DHS, they showed that the IV estimates of the effect of maternal schooling on the mortality of infants, under-5 children, and children of mothers aged 19 or younger were negative and larger in absolute value than the OLS estimates. For neonatal mortality, the IV coefficient was not statistically significant. They concluded that an additional year of maternal primary schooling reduced infant mortality by 34 % and under-5 mortality by 36 %. They also concluded that the mechanisms through which maternal education affects child survival were increased frequency of prenatal care, improved literacy levels, increased father’s educational attainment, and altered fertility behaviors.

## Pathways of Influence

Many different channels through which maternal schooling contributes to improved child survival and health have been suggested. Socioeconomic factors, such as income and wealth, are the most consistently and frequently studied mechanisms (Alemayehu Azeze and Huang 2014; Cleland and Van Ginneken 1988; Desai and Alva 1998; Frost et al. 2005; Grépin and Bharadwaj 2015; Keats 2016). However, maternal education may also increase children’s health outcomes through a range of other factors, such as attitudes, knowledge, and women’s power. Drawing on empirical studies, we identify six major pathways through which schooling may influence child survival. The proximate variables are not mutually exclusive, and this is not an exhaustive list of all potential mechanisms but rather a preliminary explanatory model to be built upon in future research.

The first pathway concerns socioeconomic position. Evidence from Uganda suggests that more-educated women are likely to have better jobs and more wealth (Keats 2016). Uneducated people tend to work in the informal market, whereas more-educated workers in the formal sector receive higher wages (El Badaoui and Rebière 2013). ​

Second, schooling may alter attitudes toward modern health services through a shift from traditional practices to acceptance of modern medicine and rational explanations of disease. Education in rural Bolivia resulted in a decrease in the use of traditional treatment practices, such as withholding fluids during diarrhea (Bicego and Boerma 1990). Basu and Stephenson (2005) argued that even slightly educated women were more likely to seek and obtain effective health care to treat their children’s illnesses, especially the common ones (which account for the bulk of children’s illnesses and mortality).

Third, education may foster personal illness control via preventive care. Results from Basu and Stephenson’s (2005) study of India suggest that completed primary education results in higher odds of various measures of personal illness control (e.g., seeking treatment for their child’s cough/fever, receiving prenatal care in pregnancy, and receiving prenatal care in the first trimester). Children of more-educated mothers in Uganda are more likely to be immunized and to receive vitamin A supplements to prevent blindness, diarrhea, and measles (Desai and Alva 1998; Keats 2016).

Fourth, schooling may affect environmental factors via good use of sanitation and hygiene practices in the home and greater access to health facilities. Previous studies (Hatt and Waters 2006; Hobcraft 1993) found that more-educated mothers are more successful at reducing the prevalence of diarrheal diseases. Moreover, women with higher levels of education tend to migrate to communities with (better) amenities and to have (greater) access to medical facilities (Desai and Alva 1998).

Fifth, schooling may facilitate the acquisition of health knowledge (Glewwe 1999). Education is related to knowledge of contraceptive methods and their more efficient use (Rosenzweig and Schultz 1989) as well as healthy behaviors during pregnancy (Grossman 1972). Agüero and Bharadwaj (2014) found that education increases the knowledge of HIV-preventive behaviors and HIV transmission in Zimbabwe.

Sixth, schooling may open new opportunities for empowerment and autonomy in the household. Increased female education has been shown to have a positive effect on women’s relative position in the household (Jejeebhoy 1992; Thomas 1990). For instance, evidence from Ethiopia suggests that education positively influences women’s participation in household decision-making (Behrman 2015).

## Universal Primary Education Reforms in Malawi and Uganda

During the 1990s, several African governments started to eliminate primary school fees (World Bank 2009). Malawi and Uganda were among the earliest countries to adopt UPE policies in sub-Saharan Africa and are the only countries for which the available data allow a comparison between women who did and did not experience free primary education and who are old enough to have completed their schooling and have had children.

In Malawi, the reform’s launch coincided with the introduction of multiparty democracy. School fees were abolished, grade by grade, starting in 1991. In September 1994, a so-called big bang approach was adopted, and free primary education was strongly enforced (Avenstrup et al. 2004). Besides the abolition of tuition fees (between US$2.35 and US$5.18 per pupil per year), uniforms were made optional, and the government paid for textbooks and exercise books (Kadzamira and Rose 2003; Rose 2002). The government also funded learning materials, classrooms, furniture, teachers’ houses, sanitation facilities, and boreholes (World Bank 2009). Primary school is made up of eight years, and the entry age is 6. In 1994, there were three school terms per year for primary schools, running generally from September to December, January to April, and April to July. The reform was implemented in September 1994, and tuition fees were abolished for all grades of primary school. Therefore, girls who were born in 1980 or earlier were not exposed to the reform, whereas girls who were born in 1981 or later were exposed to the reform.

In Uganda, a UPE policy reform was prepared in 1987, but lack of resources and political constraints impeded its implementation. The Free Primary Education reform was finally implemented in January 1997. Initially, free primary education was provided for up to four children per family, and at least two of the four children had to be girls, with priority given to disabled children. In practice, the abolition of school fees (of US$4.62 to US$7.48 per pupil per year) was applied to all children in primary school (Grogan 2006; Riddell 2003). The government assigned each school a capitation grant for instructional materials, extracurricular activities, maintenance and utilities, and administration. In addition, a facilities grant was made available to assist schools in the construction of classrooms, latrines, and teachers’ houses, and for buying furniture (Essama-Nssah et al. 2008; Grogan 2006). Primary school consists of seven years of education. The academic year starts in February and ends in December, and the entry age for primary school is 6. All children who turn 6 years old in the calendar year start school, yet the age of entry varies between ages 5 and 7 (UNESCO 2015). Therefore, girls who were born in 1983 or earlier were not exposed to the reform, whereas girls who were born in 1984 or later were exposed to the reform.

Note that using these calendar-year cut-offs would assume no grade repetition or late entry into primary school. This might seem a very strong assumption, and we found that it is, in fact, not plausible in either country: empirical evidence shows that the actual age of exit from primary school was 17 years in Malawi and 16 years in Uganda (see Fig. A1 in the online appendix). Using the same approach as Grépin and Bharadwaj (2015), we excluded women outside the official primary school age and thus only partially exposed to UPE in order to get a better picture of the schooling discontinuity. Therefore, our analysis compares the outcomes for women who were born between 1981 and 1987 with the outcomes for women who were born between 1969 and 1976 for Malawi, and the outcomes for women who were born between 1984 and 1991 with the outcomes for women who were born between 1974 and 1980 for Uganda.

Evidence from both countries indicates that the UPE reforms substantially improved enrollment. In Malawi, enrollment in primary education increased by 51 % in the first year, from 1.9 million in 1993 to 2.9 million in 1994 (Al-Samarrai and Zaman 2002; Inoue and Oketch 2008). In Uganda, it rose by 68 %, from 3.4 million in 1996 to 5.7 million in 1997, and jumped by 140 % over six years (Avenstrup et al. 2004).

Evidence also suggests that the quality of education declined because of the rapid expansion of primary school enrollment (World Bank 2009). In particular, in Malawi, the government responded to the extraordinary increase in the enrollment rate in 1994–1995 with a more than proportional increase in the number of teachers; however, about one-half of the new teachers were untrained (compared with 16 % in 1993–1994), and the other half had inadequate training (Avenstrup et al. 2004; Education Management Information Systems (EMIS) 1994; Inoue and Oketch 2008). In addition, a shortage of books, school materials, and permanent classrooms led to a more intensive use of existing facilities (Inoue and Oketch 2008; Kadzamira and Rose 2003). In Uganda, the access shock to education caused an initial decrease in various indicators, referring to the resources available to pupils. In particular, the increase in the sectoral budget in the years following the reform was not sufficient to reduce the textbook/pupil and teacher/pupil ratios. According to the national education statistics, the number of primary school teachers and classrooms, respectively, increased by only 71 % and 55 % between 1996 and 2002 (Avenstrup et al. 2004).

To accommodate the expected increase in primary school enrollment, governments in both countries opened new primary schools. The number of primary schools increased by 15 % in Malawi, from 3,216 in the 1993–1994 school year to 3,706 in 1995–1996 (EMIS 1994, 1996a); the corresponding increase in Uganda was 55 %, from 8,526 in the 1995 school year to 13,218 in 2001 (EMIS 1996b, 2001).

A notable aspect of the school construction program in both countries is that its intensity varied across districts (see the online appendix for more details). To capture the intensity of the program, we focused on the change in the number of primary schools between 1994 and 1996 in Malawi, and between 1995 and 2001 for Uganda. This measure, calculated as $\frac{\left({primaries}_1-{primaries}_0\right)}{primaries_0}$, is based on official statistics provided by the respective ministries for education (see the online appendix) and reflects the scale of the changes when the baseline level is taken into account. Previous studies have used similar approaches (Breierova and Duflo 2004; Chou et al. 2010; Duflo 2001; Osili and Long 2008). In Malawi, the mean and standard deviation of program intensity are 0.18 and 0.24, respectively; in Uganda, these are 0.65 and 0.96, respectively. Changes in the number of enrolled children in primary school were also not distributed evenly. In Malawi, the mean and standard deviation of the relative difference between 1994 and 1996 are 0.55 and 0.35, respectively; in Uganda, the mean and standard deviation of the relative difference between 1995 and 2001 are 1.35 and 0.51, respectively.

The introduction of UPE had a major impact on pupil participation and the number of primary schools. In Malawi, districts that saw the largest relative increase in the number of primary schools (i.e., high-intensity districts) experienced the largest increase in the number of enrolled children in primary school. On the other hand, in Uganda, high-intensity districts experienced an increase in the number of children enrolled in primary schools but this was not as high as that experienced by low-intensity districts. In both countries, the allocation of additional schools is weakly correlated (<.2) with the number of school-aged children—based on official statistics (EMIS 1994, 1996a, 1996b, 2001) and the 1991 Uganda census data (Minnesota Population Center 2017)—in the district before the reform. This correlation, however, is based on small samples (27 and 38 districts for Malawi and Uganda, respectively), and population density and school size vary considerably between and within districts. We could not identify a systematic allocation pattern of additional schools. Reform intensity, however, clearly varied substantially and affected enrollment, as documented in official statistics. We want to emphasize that although the quality of education deteriorated after the reform, it did not decrease more in high-intensity districts: we could not find any systematic relation between reform intensity and changes in quality of education as measured by relative differences in the pupil/teacher ratio between 1994 and 1996 in Malawi and between 1996 and 2000 in Uganda (EMIS 1994, 1996a, 1996b; Uganda Bureau of Statistics 2002a).

## Empirical Strategy

### Data

The individual-level data are from the DHS (see the online appendix for additional information). For Malawi, we used pooled data from the 2000, 2004, and 2010 DHS. For Uganda, the DHS surveys from 2000–2001, 2006, and 2011, and the DHS–Malaria Indicator Survey (MIS) from 2009 were pooled. Although all the individual surveys we selected were designed to be nationally representative, the actual sampling procedures vary between surveys, which may introduce uncertainty about the extent to which the final sample is nationally representative. However, we cannot be sure about the size or direction of the potential bias. Pooling is necessary to allow for adequate sample sizes of women of all ages for our design. In order to consider only those women who had completed their education, we included women who were at least 18 or 19 years old when the survey was conducted in Malawi and Uganda, respectively. The percentage of women who continue their studies after age 18 or 19 is lower than 15 % in both countries (see the online appendix for calculations). In addition, we excluded all children who were not living with their mothers at the time of the survey. We also excluded visitors to the household. Table 1 shows the descriptive statistics of the pooled samples.^1^

**Table 1:** Sample characteristics

	Overall		Treatment		Control	
	Mean	SD	Mean	SD	Mean	SD
A. Malawi (treatment: born 1981–1987; control: born 1969–1976)						
Characteristics of the mother						
Years of education	4.51	3.62	5.45	3.44	3.53	3.53
Age in 1994	15.54	5.68	10.38	1.91	20.97	2.22
Sample size	15,484		7,880		7,604	
Characteristics of the child						
Child is dead	0.10	0.30	0.09	0.28	0.11	0.32
Birth year	2003.8	4.25	2005.7	3.1	2001.9	4.37
Child is female	1.50	0.50	1.51	0.50	1.50	0.50
Birth order	1.82	0.38	1.70	0.46	1.94	0.23
Sample size	23,087		11,517		11,570	
B. Uganda (treatment: born 1984–1991; control: born 1974–1980)						
Characteristics of the mother						
Years of education	5.08	3.76	5.93	3.45	4.48	3.85
Age in 1997	15.86	5.18	10.24	2.10	19.88	2.03
Sample size	8,319		3,455		4,864	
Characteristics of the child						
Child is dead	0.09	0.28	0.07	0.26	0.10	0.30
Birth year	2005.0	4.21	2007.6	2.36	2003.2	4.27
Child is female	1.50	0.50	1.50	0.50	1.50	0.50
Birth order	1.79	0.41	1.65	0.48	1.89	0.32
Sample size	13,779		5,514		8,265	

*Notes:* Means are based on weighted data. Sample sizes are unweighted.

**Fig. 1:**
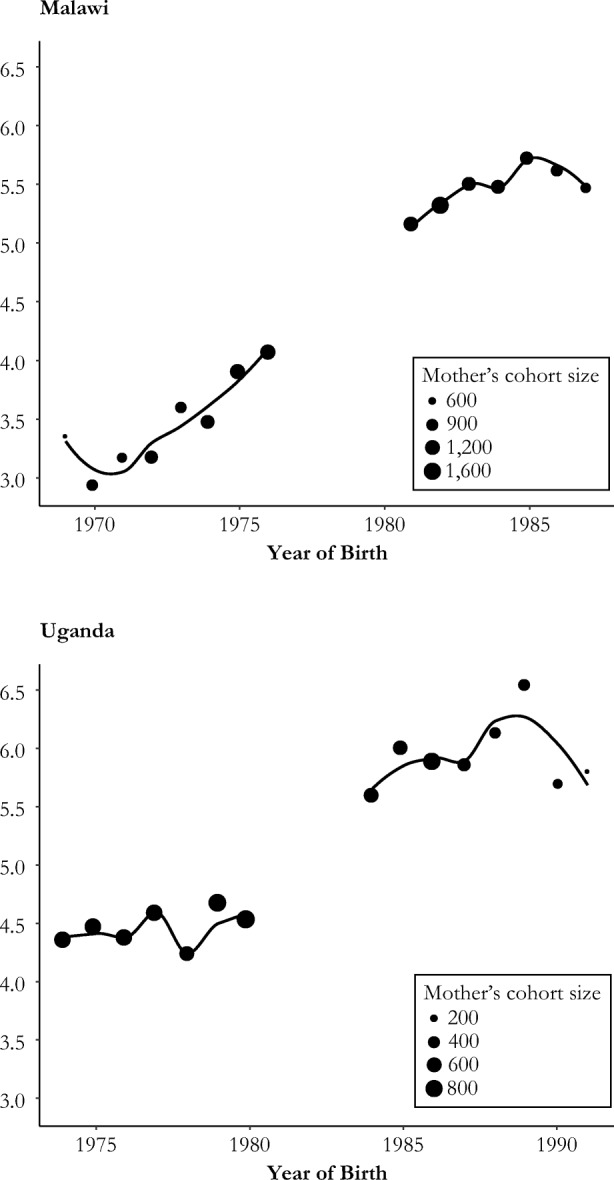
Average years of education by mother’s year of birth (averages and polynomial trend lines)

Figure 1 charts educational attainment by mother’s year of birth. On average, treated women in Malawi and Uganda had more education than untreated women. (See Fig. A2 in the online appendix for further evidence of the differences in educational attainment.)

**Fig. 2:**
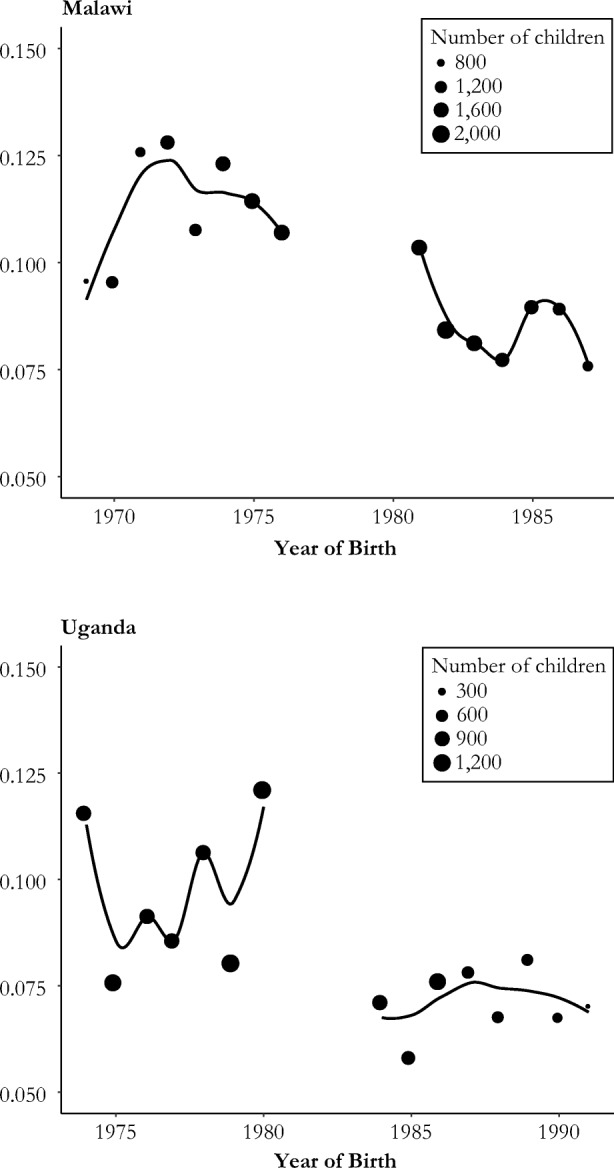
Child mortality by mother’s year of birth (averages and polynomial trend lines)

Figure 2 shows the mortality of children under age 5 born to all women in our sample, by mother’s year of birth. In both countries, mortality was lower for children of treated mothers.

### Instrumenting Maternal Education

We took advantage of the timing of the UPE reform to instrument maternal education using exposure to the reform. The mother’s year of birth and the administrative unit in which she started and completed primary school jointly determined exposure to the reform.

Using information on districts assumes that women living in a particular district at the time of the interview also had acquired their education in that district. DHS does not collect information on the district where women started and completed primary school or on where they were born. Thus, if women had changed their district of residence since primary school age, we were unable to assign an exact value of program intensity. We used current district of residence as a proxy for district of education, under the assumption that women had not moved since they started primary school. This seems to be a plausible assumption: the last censuses in Malawi (2008) and Uganda (2002) showed that internal migrants accounted for, respectively, 16 % and 13 % of the total population of the country (National Statistical Office 2009; Uganda Bureau of Statistics 2002b). In the Results section, we provide further evidence that using district of residence did not affect our results and conclusions. Nevertheless, available data did not allow us to analyze and control for fostering during childhood. Child fostering is a common childcare practice across sub-Saharan Africa and partly serves as a mechanism for households to enhance schooling opportunities for children (Lloyd and Desai 1992). It could affect our results if women were sent to live in a different household in a different district when they were school-aged. We should consider this limitation when interpreting our results.

The combined effect of the UPE program, through the increase in number of primary schools and elimination of primary school tuition fees, provided a quasi-natural experiment that allowed us to instrument maternal schooling and evaluate its impact on under-5 mortality. The first-stage regression model that we estimated for years of schooling attained by women in cohort *a* (with the oldest cohort as the omitted cohort) and district *k* reads as follows:

1 $$ {S}_{iak}={\sum}_a{C}_a{\upgamma}_a+{\sum}_k{X}_k{\upalpha}_k+{\sum}_a\left({C}_a{P}_k\right){\upbeta}_a+{\sum}_a\left({C}_a{E}_k\right){\updelta}_a+{\sum}_a\left({C}_a{N}_k\right){\uptheta}_a+{\mathbf{R}}_{iak}\boldsymbol{\upeta} +{\upnu}_{iak}, $$

where *S*_*iak*_ is the endogenous variable, comprising years of education of woman *i*, born in year *a* and district *k*; *C*_*a*_ is a dummy variable for cohort *a*; *X*_*k*_ is a dummy variable for district *k*; *P*_*k*_ is program intensity in district *k*; *E*_*k*_ is the number of girls in primary school before UPE in district *k*; *N*_*k*_ is the number of primary school–aged children before UPE in district *k*; **R**_*iak*_ is the categorical variable religion (Catholic, Presbyterian, Muslim, other Christian, no religion, and other; not available for Uganda); and ${\upnu}_{iak}$ is the error term. By including the number of primary school–aged girls enrolled before UPE and the number of primary school–aged children before UPE, each interacted with the cohort dummy variables, we controlled for time- and district-varying factors correlated with pre-program enrollment and captured yearly and district differences in the demand for education.

**Fig. 3:**
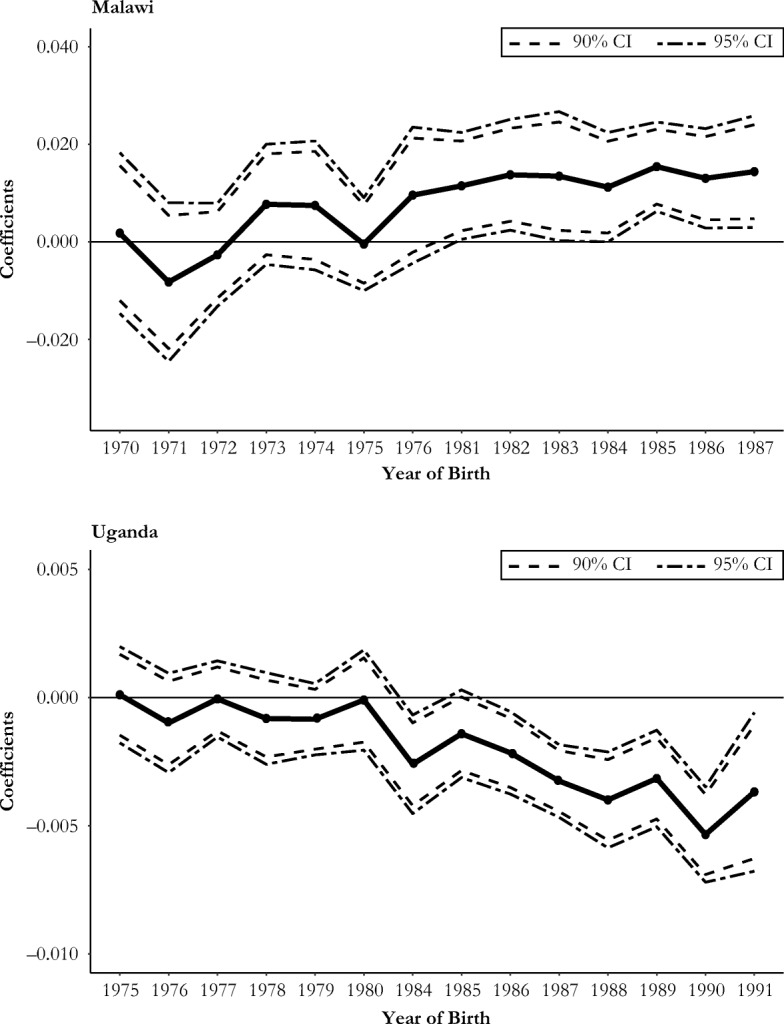
Coefficients of the interactions year of birth × program intensity in the district of residence in (Eq. 1)

Panel a of Fig. 3 shows the coefficients and the confidence intervals of the interactions between year of birth and program intensity in the woman’s district of residence in Malawi. Panel b illustrates the corresponding coefficients in the Uganda equation. In both countries, the coefficients are 0 for the oldest cohorts and are statistically different from 0 for the youngest cohorts. These results suggest that the program intensity effects were restricted to the treatment group and that cohorts in the control group were not affected by the program.

The *F* ratio of the test—in which the coefficients of the interactions between year of birth and program intensity for the youngest cohorts are statistically significant as a set—is 32.2 for Malawi and 93.9 for Uganda. Because the coefficients of the interactions between year of birth and program intensity are 0 for the oldest cohorts, and different from 0 for the youngest cohorts, we could reduce the number of program intensity coefficients that must be estimated by imposing a simple restriction. This can reduce the degree of multicollinearity among the independent variables and improve the precision and efficiency of the estimates of the effect of the program. The model reads as follows:

2 $$ {S}_{iak}={D}_a\upgamma +{\sum}_k{X}_k{\upalpha}_k+\left({D}_a{P}_k\right)\upbeta +\left({D}_a{E}_k\right)\updelta +\left({D}_a{N}_k\right)\uptheta +{\mathbf{R}}_i\boldsymbol{\upeta} +{\upnu}_{iak}, $$

where *D*_*a*_ is a dummy variable taking the value 1 if the mother is in the treatment group, and 0 otherwise.

### Cox Specification

In linear models, the 2SLS approach is used to address endogeneity by replacing the endogenous value with the predicted value for education from the first-stage estimation. Because our outcome was risk of death up to age 5, we used a survival model to account for the right-censoring of the data (Allison 1982). When survival models are used, the two-stage residual inclusion (2SRI) approach, which is identical to the 2SLS in a linear setting, has been shown to yield consistent estimates (Atiyat 2011; Terza et al. 2008). Unlike the 2SLS, in the second stage of the 2SRI regression, both the first-stage residuals, ${S}_{v_i}$, and the endogenous variable, *S*_*i*_, are included in the model to be fitted. We estimated a Cox proportional hazards model for right-censored data^2^ (Cox 1972):

3 $$ h(t)={h}_0(t){e}^{\left({\sum}_k{X}_k{\upalpha}_k+{S}_i{\uprho}_1+{S_v}_i{\uprho}_2+\left({D}_a{E}_k\right)\updelta +\left({D}_a{N}_k\right)\uptheta +{\mathbf{R}}_i\boldsymbol{\upeta} +{G}_j\uppi +{B}_j\uptau +{\sum}_y{C}_y{\uplambda}_y+{\upvarepsilon}_{jyak}\right)}, $$

where *h*_0_(*t*) is an unspecified baseline hazard function, *G*_*j*_ is child sex, *B*_*j*_ is child birth order, and *C*_*y*_ is a dummy variable for child cohort *y*. We controlled for child birth order with indicator variables for (1) first birth and (2) second birth and more. Child cohort was represented by indicator variables for born in Malawi in 1995–1999, 2000–2004, and 2005–2010, and for born in Uganda in 1995–2000, 2001–2006, and 2007–2011. Given the timing of the surveys and our sample selection (i.e., births occurred five years prior to the survey), child cohort further captured potential effects from pooling data across surveys. All coefficients are log hazard ratios, and ρ_1_ is a consistent estimate for the true effect of maternal education on under-5 mortality. Therefore, exp(ρ_1_) is the hazard ratio associated with a one-year increase in maternal education, and (exp(ρ_1_) − 1) is the effect of an additional year of maternal schooling on the probability of dying before age 5 for children of compliers (i.e., mothers going to primary school if eligible, and not going if not eligible). If exp(ρ_1_) is smaller than 1 and statistically different from 0, there is a causal negative relationship between maternal education and under-5 mortality. The ρ_2_ is the effect of the first-stage residuals on under-5 mortality; its interpretation is equivalent to that of the Wu-Hausman test in a 2SLS framework, wherein a statistically significant coefficient indicates endogeneity in the relationship between maternal education and under-5 mortality. Moreover, the Cox model assumes that the hazards are proportional over time. Proportionality of the effect of years of education was confirmed by testing the slope of Schoenfeld residuals. Furthermore, simulations have shown that unadjusted standard errors are accurate when the 2SRI approach is used (Atiyat 2011:27).

To quantify the magnitude of the effect of maternal schooling, we estimated the population attributable fraction (PAF) of under-5 mortality associated with maternal schooling (Chen et al. 2010)—that is, the fraction of under-5 mortality cases that would not have occurred if mothers had had some education. In the presence of confounders, **W**, we used the following formula:

4 $$ PAF=\frac{\mathrm{pr}\left(D=1\right)-{\sum}_{k=1}^m pr\left(\mathbf{W}={\mathbf{w}}_k\right) pr\left(D=1|Z=0,\mathbf{W}={\mathbf{w}}_k\right)}{\mathrm{pr}\left(D=1\right)}, $$

where *D* is the binary status variable (alive or dead), *Z* is a binary exposure indicator, and **w**_1_, . . . , **w**_*m*_ are the *m* levels of **W**. In our case, where the exposure variable is continuous, an analogous formula involves integration of the exposure level distribution. To calculate excess deaths associated with low maternal education, the PAF is then multiplied by the total number of under-5 deaths (data U.N. IGME, 1990–2016) in Malawi occurring in the 1995–2010 birth cohort in 2003; and in Uganda, occurring in the 1995–2011 birth cohort in 2004, which was the mean year of death in the samples.

### Pathways

We studied six pathways through which maternal education might reduce child mortality. We used the 2SLS strategy and regressed the pathway indicators on the predicted value of maternal education from the first stage. Table 2 gives descriptive statistics for the pathway indicators.

**Table 2:** Summary statistics of the pathway indicators

	Overall		Treatment		Control	
	Mean	SD	Mean	SD	Mean	SD
A. Malawi (treatment: born 1981–1987; control: born 1969–1976)						
Socioeconomic status						
Wealth index (0 to 5.1)	0.73	0.60	0.75	0.64	0.71	0.56
Medical care: money (*n* = 12,010)	0.42	0.49	0.46	0.50	0.37	0.48
Attitudes toward modern health services						
Use of modern contraception (*n* = 13,427)^a^	0.42	0.49	0.43	0.49	0.41	0.49
Personal illness control						
Personal illness control (0 to 8) (*n* = 15,274)^b^	3.73	0.78	3.72	0.72	3.74	0.83
Environmental characteristics						
Close to health facility (*n* = 12,010)	0.41	0.49	0.43	0.49	0.37	0.48
Health knowledge						
Knowledge about contracting AIDS (0 to 2.5) (*n* = 15,042)	1.96	0.67	1.98	0.66	1.93	0.66
Knowledge about transmission of AIDS (0 to 2.5) (*n* = 15,042)	1.98	0.64	2.02	0.60	1.95	0.67
Knowledge about ovulation (0 to 1) (*n* = 15,465)	0.44	0.33	0.45	0.32	0.44	0.34
Empowerment						
Decision-making (0 to 2.5) (*n* = 14,491)	0.92	0.81	0.91	0.80	0.92	0.83
Empowered domestic violence (0 to 3.6) (*n* = 14,491)	3.19	0.92	3.24	0.87	3.13	0.96
B. Uganda (treatment: born 1984–1991; control: born 1974–1980)						
Socioeconomic status						
Wealth index (0 to 3.9)	1.21	0.72	1.40	0.65	1.07	0.74
Medical care: money (*n* = 6,827)	0.41	0.49	0.45	0.50	0.38	0.49
Attitudes toward modern health services						
Use of modern contraception (*n* = 5,661)^a^	0.27	0.44	0.25	0.43	0.28	0.45
Personal illness control						
Personal illness control (0 to 7.3) (*n* = 6,679)^b^	2.69	0.77	2.71	0.70	2.68	0.82
Environmental characteristics						
Close to health facility (*n* = 6,827)	0.51	0.50	0.52	0.50	0.50	0.50
Health knowledge						
Knowledge about contracting AIDS (0 to 2.6) (*n* = 6,637)	2.16	0.71	2.18	0.71	2.15	0.71
Knowledge about transmission of AIDS (0 to 2.1) (*n* = 6,637)	1.42	0.67	1.41	0.68	1.43	0.66
Knowledge about ovulation (0 to 1) (*n* = 6,831)	0.43	0.33	0.42	0.32	0.43	0.33
Empowerment						
Decision-making (0 to 2.4) (*n* = 6,076)	1.22	0.84	1.17	0.84	1.25	0.83
Empowered domestic violence (0 to 2.7) (*n* = 6,076)	1.57	0.88	1.64	0.89	1.53	0.87

*Notes:* Means are based on weighted data. Sample sizes are unweighted.

^a^Excluding pregnant women.

^b^Excluding less recent births.

We used two indicators for socioeconomic status, the first pathway: (1) the DHS comparable wealth index, based on ownership of durable goods and quality of housing; and (2) a binary variable indicating whether the woman did not consider money a barrier to obtaining medical care (1 = money not a barrier).

For the second pathway—attitudes toward modern health services—we used a binary variable of whether the woman used modern contraception (1 = yes).

For the third pathway—personal illness control—we used a latent variable, based on a factor analysis of three variables: (1) the number of tetanus injections received during pregnancy; (2) whether the woman had seen a health professional during pregnancy; and (3) the number of antenatal visits during pregnancy. Higher scores indicate higher personal illness control.

The fourth pathway explored the impact of maternal education through environmental factors, as measured by a binary variable of whether the distance to a health facility was not a big problem (1 = not a big problem).

The fifth pathway—health knowledge—was measured by three indicators: knowledge about contracting AIDS, knowledge about transmitting AIDS, and knowledge about the ovulatory cycle (see the online appendix for exact questions). Three questions captured knowledge about contracting AIDS, two questions were related to knowledge about its transmission, and a single question tested the women’s knowledge about ovulation. Factor analysis showed that the five questions on AIDS loaded on two factors, with an eigenvalue larger than 1. Higher scores mean greater health knowledge.

Finally, we used two indicators for the sixth pathway, women’s empowerment. Eight questions capturing women’s empowerment were consistently available across DHS surveys. Based on a factor analysis showing two factors with an eigenvalue greater than 1, we distinguished two indicators. The first, decision-making, used three questions about the husband’s and wife’s roles in decision-making in the household with regard to woman’s health care, large household purchases, and visits to family and friends. The second indicator, empowered domestic violence, was based on five questions on whether a man is justified in beating his wife/partner under various circumstances: if she (1) went out without telling him; (2) neglected the children; (3) argued with him; (4) refused to have sex with him; and (5) burned the food. The five items were coded as 1 if the man is not viewed as justified, and 0 otherwise. Higher scores on both indicators represent higher empowerment for women.

## Results

### Exposure to UPE and Maternal Education

Did the UPE reform affect maternal education? Table 3 shows the effects of UPE on maternal education in Malawi and Uganda. In Malawi, gains in educational attainment were larger in high-intensity districts compared with low-intensity districts. In Uganda, however, educational attainment increased more in high-intensity districts compared with low-intensity districts. The estimates suggest that a 1 percentage point increase in reform intensity is associated with an increase in years of education for the youngest cohorts of 1.7 (i.e., 1.644 + 0.01) and 1.5 (i.e., 1.490 – 0.002) years in Malawi and Uganda, respectively.

**Table 3:** First-stage results: Impact of UPE on maternal education

	Years of Education	
	Malawi	Uganda
Young	1.644***	1.490***
	(0.214)	(0.318)
Young × Program Intensity	0.0109***	–0.00228***
	(0.003)	(0.0004)
Kleibergen–Paap *rk* Wald *F* Statistic	57.105	23.261
*N*	15,484	8,319
*R* ^2^	.710	.717

*Notes:* The standard errors, shown in parentheses, are clustered at the district level. All regressions include religion (Malawi only), district fixed effects, interaction between the dummy variable for young and the district-specific number of girls in primary school before UPE, and interaction between the dummy variable for young and the number of primary school–aged children before UPE. The variable program intensity is expressed as percentage.

***p < .001

For UPE exposure to be used to instrument for maternal schooling, it must be strongly correlated with maternal years of schooling. An instrument is considered relevant if the *F* statistic on the excluded instrument in the first stage is greater than 10 (Staiger and Stock 1997); however, this rule of thumb is invalid when multiple instruments are used in the first-stage regression. In this case, two more powerful tests are the Cragg–Donald Wald *F* statistic and its robust counterpart, the Kleibergen–Paap Wald *rk F* statistic. Stock and Yogo (2005) compiled critical values for these statistics for 2SLS estimators: if the test statistics are below these critical values, the instruments are considered weak. We tested the strength of the set of instruments by comparing the Kleibergen–Paap Wald *rk F* statistic with the Stock and Yogo (2005) critical value of 19.93 for 10 % maximal test size distortion.^3^ The *F* statistics of 57.1 and 23.3 indicate that our instruments are not weakly correlated with schooling.

The use of district of residence as a proxy for district of birth introduced concerns about migration. Was district of residence exogenous to the reforms? Cross-district migration patterns in census data suggest that among districts with high internal migration rates, those experiencing a net gain in population (i.e., having more immigrants than emigrants) were both high- or low-intensity districts and that districts experiencing a net loss in population (i.e., having more emigrants than immigrants) were both high- or low-intensity districts (National Statistical Office 2009; Uganda Bureau of Statistics 2002b). In other words, internal migrants had not moved to high-intensity districts only, and the decision to migrate to one district rather than another was not associated with the reform. Nevertheless, two main sources of bias may arise from measurement error. One source of bias could be introduced by child fostering. In particular, if women were sent to live in a different household in higher-intensity districts during childhood, we might have overestimated the value of β from (Eq. (2)). We could not control for this mechanism in our analysis, and this might limit our results. Furthermore, women might have migrated after completing primary education, and this might have resulted in some bias in our β estimate from (Eq. (2)). In particular, if women had migrated to higher-intensity districts, we might have underestimated the value of β, the opposite being true if women had migrated to lower-intensity districts. The districts with a sizable share of immigrants were Lilongwe City (Malawi) and Kalangala (Uganda): 52 % and 38 % of their populations, respectively, migrated from other districts. Therefore, we performed an analysis without Lilongwe City and Kalangala to account for possible measurement error. Table 4 shows that the bias in β was negligible and that the results were robust to excluding Lilongwe City and Kalangala.

**Table 4:** First-stage results: Robustness checks excluding districts with high internal migration

	Years of Education	
	Malawi Excluding Lilongwe City	Uganda Excluding Kalangala
Young	1.630***	1.517***
	(0.214)	(0.322)
Young × Program Intensity	0.0108***	–0.00227***
	(0.003)	(0.0004)
Kleibergen–Paap *rk* Wald *F* Statistic	54.530	23.121
*N*	15,167	8,298
*R* ^2^	.708	.716

*Notes:* The standard errors, shown in parentheses, are clustered at the district level. All regressions include religion (Malawi only), district fixed effects, interaction between the dummy variable for young and the district-specific number of girls in primary school before UPE, and interaction between the dummy variable for young and the number of primary school–aged children before UPE. The variable program intensity is expressed as percentage.

***p < .001

### Maternal Education and Child Mortality

We now move to the effect of maternal education on child mortality. We estimated two Cox models for each country: one model treating maternal schooling as exogenous, and another estimated using the 2SRI approach that treats maternal education as endogenous. Table 5 shows the results of the impact of maternal education on child mortality.

**Table 5:** Second-stage results: Maternal education and child mortality

	Child Is Dead			
	Malawi		Uganda	
	Non–2SRI	2SRI	Non–2SRI	2SRI
	Odd Ratio (95 % CI)	Odd Ratio (95 % CI)	Odd Ratio (95 % CI)	Odd Ratio (95 % CI)
Years of Education	0.971***	0.900*	0.951***	0.834^†^
	(0.958–0.984)	(0.811–0.998)	(0.934–0.969)	(0.687–1.012)
First-Stage Residuals		1.080		1.142
		(0.973–1.199)		(0.941–1.385)
Overidentification Test (*p* value)		.1		.7
*N*	23,087	23,087	13,779	13,779

*Notes:* The overidentification value is the *p* value of the overidentification test. All regressions include religion (Malawi only), child sex, birth order, child year of birth fixed effects, district fixed effects, interaction between the dummy variable for young and the district-specific number of girls in primary school before UPE, and interaction between the dummy variable for young and the number of primary school–aged children before UPE.

^†^*p* < .10; *p < .05; ***p < .001

In line with the literature, the simple Cox model suggests that increased female education had a negative impact on child mortality in both Malawi and Uganda (columns 1 and 3, Table 5). In particular, in Malawi, the odds of dying for children of women with one additional year of education were 2.9 % lower (column 1). The model in column 2 accounts for the endogeneity of education and shows that the effect of maternal education on child mortality was even larger, with a one-year increase in education significantly reducing the probability of dying before age 5 by about 10.0 %. In Uganda, the odds of dying for children of women with one additional year of education were 4.9 % (column 3). Here, too, the causal relationship between maternal education and child mortality was even stronger when we controlled for the endogeneity of the education variable: for each additional year of education, children had a 16.6 % lower probability of dying (column 4). The first-stage residuals had a positive impact on under-5 mortality, revealing that more-educated women had, on average, unobservable characteristics that were associated with a higher level of child mortality (columns 2 and 4) (i.e., negative selection). The effect, however, is not statistically significant (*p* value of ρ_2_ is .15 for Malawi and .18 for Uganda), which indicates both that maternal education was not endogenous and that the conventional Cox estimator was consistent. These results further indicate that the conventional Cox estimate is smaller than the 2SRI Cox estimate, although not significantly, which suggests that omitted variable bias was not a significant factor.

Furthermore, as a robustness check, we performed the same analysis excluding Lilongwe City (Malawi) and Kalangala (Uganda). Table 6, columns 1 and 3, indicate that the results are robust to excluding Lilongwe City and Kalangala. In addition, for the sake of comparing our results with those in the three previous studies analyzing the relationship between maternal education and child mortality (Keats 2016; Makate 2016; Makate and Makate 2016), we specified a linear model estimation for under-5 mortality (i.e., we used a 2SLS approach). The findings show that the negative impact of maternal education on under-5 mortality lost statistical significance in both countries (Table 6, columns 2 and 4). These findings indicate that ignoring the right-censoring issue of the data can lead to misleading conclusions.

**Table 6:** Second-stage results: Robustness check and linear specification

	Child Is Dead			
	Malawi		Uganda	
	2SRI Excluding Lilongwe City	2SLS^a^ Full Sample	2SRI Excluding Kalangala	2SLS^a^ Full Sample
	Odd Ratio	Linear Coefficient	Odd Ratio	Linear Coefficient
	(95 % CI)	(SE)	(95 % CI)	(SE)
Years of Education	0.897*	–0.008	0.846^†^	–0.011
	(0.807–0.996)	(0.006)	(0.698–1.026)	(0.007)
First-Stage Residuals	1.083		1.126	
	(0.974–1.204)		(0.928–1.365)	
Overidentification Test (*p* value)	.1	.2	.7	.6
*N*	22,635	23,087	13,741	13,779

*Notes:* The overidentification value is the *p* value of the overidentification test. All regressions include religion (Malawi only), child sex, birth order, child year of birth fixed effects, district fixed effects, interaction between the dummy variable for young and the district-specific number of girls in primary school before UPE, and interaction between the dummy variable for young and the number of primary school–aged children before UPE.

^a^The coefficient is the linear effect of an additional year of maternal schooling on child mortality. The adjusted standard errors, shown in parentheses, are clustered at the district level.

^†^*p* < .10; *p < .05

We now turn to the possibility that our instruments are correlated with under-5 mortality. The exclusion restriction assumption says that the reform did not affect under-5 mortality directly, only through its effect on maternal schooling. We tested this restriction following the methodology described by Bollen et al. (1995)—that is, by testing the null hypothesis that the instruments are uncorrelated with the error term in the second stage. The test involves including all but one excluded variable in the equation that controls for endogeneity of maternal education and testing that the variable is a significant predictor (Bollen et al. 1995). A rejection of the null hypothesis would cast doubt on the validity of the instruments as well as on the quality of the second-stage estimates. The test statistic failed to reject the null hypothesis of instrument exogeneity in both countries (*p* value was .1 for Malawi and .7 for Uganda; see Table 5), pointing to the validity of the overidentifying restrictions.

We also calculated estimates of attributable mortality and excess deaths to low maternal education. Given both the loss in efficiency associated with 2SRI and the consistency of the conventional Cox estimator in both countries, we used the hazard ratios from models in columns 1 and 3 of Table 5 to calculate the PAF. The median PAF was –12.2 % (95 % confidence interval (CI): –18.1 % to –6.6 %) for Malawi and –23.9 % for Uganda (95 % CI: –33.7 % to –14.9 %). An increase in maternal education would have led to a 12.2 % and 23.9 % reduction in under-5 mortality risk in Malawi and Uganda, respectively. This translates into 8,477 (of 69,569) and 39,296 (of 164,406) excess deaths in Malawi and Uganda, respectively.

The UPE reform may have affected both the quantity and quality of education. This matters for our interpretation of the effect of education on child mortality. In particular, if the quality of schooling had increased after the reforms, this would affect our interpretation of ρ_1_ from (Eq. (3)). If UPE significantly affected both the quantity and quality of education, ρ_1_ would still capture the effect of the reform but not the effect of an additional year of education. However, we found no evidence that the quality of education provided improved after the reforms. In fact, it seems more likely that education quality indicators decreased for Malawi and Uganda in the late 1990s and early 2000s (Avenstrup et al. 2004). To examine whether UPE affected quality of education, we compared the returns to education for the oldest cohorts in our treatment group (i.e., Treatment 1 (T1): 11–13 years old at UPE) with the returns for the youngest cohorts in our treatment group (i.e., Treatment 2 (T2): 8–10 years old at UPE) with the returns for younger cohorts (i.e., Treatment 3 (T3): 5–7 years old at UPE). Individuals were differently affected by UPE: compared with women in T1, those in T2 were exposed to the reform longer, and those in T3 were exposed to the program since the beginning of their education. If quality of education had increased, we would expect the effects of education on child mortality to be statistically different. The coefficients for T1, T2, and T3 are not statistically different from one another, however. Values of the *t* test of the difference in the education coefficients between T1 and T2, between T1 and T3, and between T2 and T3 are –0.05, 1.2, and 1.3 in the Malawian sample; these values for the Ugandan sample are –0.4, –1.2, and –0.9. This finding suggests that the UPE policy affected child mortality mainly through the quantity of education.

### Maternal Education and Pathways of Influence

Next we examined various mechanisms through which maternal education might reduce child mortality. These mechanisms are grouped into broader categories in Table 7 (see the online appendix for reduced-form estimates). With regard to socioeconomic position, schooling had a significant positive effect on wealth in Uganda only, and having more education increased the probability of not considering money to be a barrier to medical care in both countries.

**Table 7:** Second-stage results: Maternal education and 10 pathways indicators (one model per indicator per country)

	Malawi	Uganda
Years of Education on	2SLS	2SLS
Socioeconomic Status		
Wealth index	0.008	0.151***
	(0.010)	(0.029)
Medical care: Money	0.029**	0.075**
	(0.010)	(0.023)
Attitudes Toward Modern Health Services		
Use of modern contraception	0.028**	0.027^†^
	(0.009)	(0.015)
Personal Illness Control		
Personal illness control	–0.004	0.079^†^
	(0.014)	(0.043)
Environmental Characteristics		
Close to health facility	0.031**	0.009
	(0.010)	(0.020)
Health Knowledge		
Knowledge about contracting AIDS	0.006	0.052
	(0.013)	(0.037)
Knowledge about transmission of AIDS	0.023*	0.001
	(0.010)	(0.029)
Knowledge about ovulation	–0.015^†^	–0.006
	(0.008)	(0.015)
Empowerment		
Decision-making	0.001	–0.066
	(0.022)	(0.045)
Empowered domestic violence	0.095***	0.204***
	(0.015)	(0.061)

*Notes:* The standard errors, shown in parentheses, are clustered at the district level. All regressions for mothers include religion (Malawi only), district fixed effects, interaction between the dummy variable for young and the district-specific number of girls in primary school before UPE, and interaction between the dummy variable for young and the number of primary school–aged children before UPE. All regressions for children further include child sex, birth order, and child year of birth fixed effects. Sample excludes missing information and Uganda DHS–MIS 2009 because these questions are not included in the DHS–MIS questionnaire (DHS restriction). First-stage results are unaffected by the reduction in the sample size.

^†^*p* < .10; *p < .05; **p < .01; ***p < .001

In both countries, schooling had a significant effect on the odds of use of modern contraceptive methods. The reduced-form results confirm that exposed women were more likely than non-exposed women to use modern contraceptive methods. With regard to personal illness control, increased education had a significant effect in Uganda only.

For the environmental pathway, exposure to the reform was associated with increased proximity to a health facility. In Uganda, the effect was not statistically significant.

Did education affect health knowledge? For Malawi, schooling increased knowledge about the transmission of AIDS, but there was no significant effect for Uganda. The reduced-form results confirm that exposed women had more knowledge about the transmission of AIDS. Counterintuitively, maternal schooling decreased correct knowledge about ovulation in Malawi; however, it is not clear that the given answer categories allowed for a clear distinction and weighting between correct, semi-correct, and incorrect answers (see the online appendix). Therefore, we should be very cautious in generalizing such a specific result.

Finally, we examined whether female empowerment was shaped by schooling. In both countries, schooling had no significant effect on women’s participation in household decision-making; however, more-educated women were more likely to reject domestic violence. The reduced-form results suggest that exposed women were more likely than non-exposed women to reject domestic violence.

## Conclusions

We investigated the causal effect of maternal education on child mortality by taking advantage of educational reforms that introduced free primary education in Malawi and Uganda in 1994 and 1997, respectively. In both countries, the reform caused an increase in maternal schooling. We modelled child mortality using a Cox model to take censoring into account. Findings from these models suggest that each additional year of education caused a 10.0 % and 16.6 % lower probability of dying before age 5 in Malawi and Uganda, respectively. These estimates are higher than, but not significantly different from, conventional estimates. Therefore, our findings do not support the idea that the relationship between maternal education and under-5 mortality is biased upward as a result of omitted variables. These findings are in line with those of similar studies exploiting exogenous variation in education in developing countries, which obtained IV estimates that were greater than OLS estimates (Breierova and Duflo 2004; Chou et al. 2010; Grépin and Bharadwaj 2015; Makate and Makate 2016). Our PAF estimates (of 12 % and 24 % for Malawi and Uganda, respectively) are consistent with the range of estimates reported in these studies (i.e., PAFs of 8 % to 36 %).

Our findings differ from those reported by Keats (2016) and Makate (2016), however, who found a nonsignificant effect of maternal education on child mortality. There are two main possible explanations for this inconsistency. First, these prior studies used different samples, which could have biased their estimates. In particular, Keats (2016) considered only firstborn children, and Makate (2016) instead analyzed all fully exposed births. As discussed earlier, using different samples leads to selection bias. Previous studies have shown that birth-history data in sub-Saharan African DHS surveys can suffer from omission and backward displacement of births, and they are thus vulnerable to the underreporting of births and deaths of children for five years before the survey (Pullum et al. 2013; Schoumaker 2011); this is particularly true for less-educated mothers (Schoumaker 2011). Second, Makate (2016) and Keats (2016) used a linear specification in the second-stage model; we used a Cox model, and this leads to different results.

We also assessed whether quality of education might have influenced our results, but we found that the UPE policy affected child mortality mainly through quantity of education. Despite the established causal relationship, we did not question whether the quality of education was unnecessary in triggering demographic change. The UPE policy was meant to increase the quantity of education, and this analysis considered only the effects of the expansion in school participation and, in doing so, tried to isolate those effects. The lack of detailed information about the efficiency of the two reforms, local administrative wastage, and corruption—and their district variation—limits our understanding of the role of quality of education and its relationship with reform intensity, which could introduce some bias in the modelling. More in-depth studies on the *quality* of education are needed to examine how the causal effect was brought about.

Our study provides evidence of the potential mechanisms underlying the impact of maternal education on child mortality. We showed that financial barriers to medical care, attitudes toward modern health services, and rejection of domestic violence may play a role. Moreover, being more educated seems to have enhanced proximity to a health facility and knowledge about the transmission of AIDS in Malawi, and wealth and improved personal illness control in Uganda. We could explore only a limited number of variables in DHS, and we had to rely on very specific indicators for each pathway. Hence, we have to be careful in generalizing the results. Where we do not find evidence for a pathway, the lack of evidence might be due to the particular way of measuring the pathway. Further studies are needed to examine the causal relationship between education and possible mediating variables in more detail. In particular, more work is required to understand how health knowledge is shaped by education.

We focused exclusively on maternal education, thus ignoring paternal education, even though it may be important, too (Chou et al. 2010). However, we had only one reform to instrument education with, and instrumenting two variables in one model was not feasible. Moreover, it is well known that the education of husbands and wives is strongly correlated (Smits et al. 1998), which would complicate identifying any independent effects. Also, including fathers would have reduced the sample size, and it is not always certain that the woman’s partner is actually the child’s father. Nevertheless, we acknowledge that the estimates for maternal education might be overestimated due to omission of the variable paternal education (Breierova and Duflo 2004; Chou et al. 2010).

To conclude, our findings show that the reforms in Malawi and Uganda were effective in increasing maternal education and, as a consequence, in decreasing child mortality. They suggest that in other sub-Saharan countries, which have more recently started to eliminate primary education fees as part of the UNESCO Education for All initiative, free primary education for girls could have similar implications for reducing child mortality. Finally, the UPE policy induced variation in access to schooling only at the primary school level; however, the effect of more schooling at the secondary level may be different. In 2007, the government of Uganda was the first in sub-Saharan Africa to implement a free universal secondary education policy. This reform provides an important opportunity to assess the causal effect of continued education and address some of the aforementioned limitations and remaining questions.

## Electronic supplementary material

ESM 1 (DOCX 382 kb)

## Footnotes

1 Descriptive statistics were weighted by DHS sample weights.

2 The Cox proportional hazards model is preferred over a discrete-time event history model, which is widely used to estimate child mortality. The exogeneity of excluded instruments is hard to assess in the second model given its complex structure.

3 The Stock–Yogo test of maximal bias is not available unless the number of overidentifying restrictions is at least 2.
