# Supplementary material for: The Causal Effect of Maternal Education on Child Mortality: Evidence From a Quasi-Experiment in Malawi and Uganda
Source: Demography. 2019 Oct 7;56(5):1765–90. doi: 10.1007/s13524-019-00812-3 (PMC6797651; doi:10.1007/s13524-019-00812-3)
Supplement: Supplementary file 1 — (DOCX 382 kb) [file 13524_2019_812_MOESM1_ESM.docx]

# **Online Appendix**

**Treatment and control groups: Malawi and Uganda**

When examining the role of women’s education on fertility and child health outcomes in Uganda, Keats (2016) considered women aged 7–14 in 1997 as the treatment group and women aged 15–22 in 1997 as the control group. Keats justified this by arguing that previous studies (Grogan 2006, 2009) have proved that UPE in Uganda had an impact on school starting age: UPE increased the probability of a child entering school before age 8 by 9 %. Furthermore, Breglia et al. (2008) and Tamusuza (2011) found that children attended primary school when they were older than the officially recommended age as a result of grade repetition, long-term absenteeism, and late entry into school, and that the age of exit from primary school was 16 years (Tamusuza 2011:125). A similar argument applies to Malawi: empirical evidence suggests that 15 % of girls who were 14 years old (i.e., the official age of exit from primary school) were in standards (years) 1 to 5 instead of standard 8 (EMIS 2004:17).

Using the 1992 Malawi DHS and the 1995 Uganda DHS Household Surveys, we found that the primary school attendance of girls starts to decline considerably at the age of 16 years in Malawi and 15 years in Uganda (Fig. A1), which suggests that the actual age of exit from primary school was effectively 17 years in Malawi and 16 years in Uganda.

**The administrative structure of the educational sector in Malawi and Uganda**

Malawi is divided into three administrative regions: Northern, Central and Southern. In 1994, there were twenty-four districts, five in the Northern Region, nine in the Central Region, and ten in the Southern Region; and in each district there are Traditional Authorities and Villages. The provision of primary schooling and primary school teacher training was a responsibility of the Ministry of Education, Science and Technology. However, the administrative structure of education was assigned to six education divisions, namely Northern, Central Eastern, Central Western, South Eastern, Shire Highlands, and South Western, and to the respective 28 district education offices (Chimombo et al. 2000; EMIS 2006). The latter included 24 districts and 4 urban cities (Mzuzu city, Lilongwe urban, Zomba urban, and Blantyre urban). Because in the 2000 and 2010 DHSs Mzuzu city and Mzimba district were combined, we could identify 27 districts instead of 28.

In 1996, Uganda was divided into four statistical (not administrative) regions: Central, Eastern, Northern and Western. The country was further divided into thirty-nine administrative districts, which were subdivided into counties, sub-counties and parishes (and sub-parishes in most cases). Under the local Government Act of 1997, public services such as nurseries, primary schools, special schools, and technical schools fell under the administration and management of District Councils. These councils had the authority to formulate, approve, and execute their own development plan, register UPE children, and distribute textbooks. Moreover, monthly remittances for schools from central government were transmitted to local government via the district administration officer, who in turn passed them on to schools (Uganda Ministry of Education and Sports 2004 cited in Nakabugo et al. (2008); Nambalirwa (2010)). Because in the 2000–2001 DHS Ntungamo district was combined with Mbarara district, we could identify 38 districts instead of 39. We used GPS data to identify the location of households within districts.^[[1]](#footnote-1)^

**Education management information systems database of the Ministry of Education**

The district-level data are from the education management information systems database of the ministries of education of Malawi and Uganda. The ministries of education in both countries published data from school records submitted by primary school administrations. These data might have some limitations. It has been shown that the estimates on gross enrolment rate were different from the survey estimates either because: (1) some school did not respond to the Ministry’s request for information; (2) government school fund allocations were based on enrolment, so school administrators may have been motivated to inflate pupil numbers (Policy and Operations Evaluation Department (IOB) 2008 cited in Tamusuza 2011). However, in our analysis we used the number of actual primary schools (as opposed to the number of responding schools). Therefore, it could be that the number of primary schools was underreported, but this would likely be true in both academic years considered and thus would not substantially affect the difference in the number of schools that we consider in this paper.

Because the Uganda Ministry of Education and Sports did not collect data on actual primary schools for 1996, we used data on 1995, which illustrates the situation as of December 1995.

**Demographic and Health Surveys**

The DHS are national household sample surveys measuring population, health, socioeconomic and, for most of them, anthropometric indicators, which emphasize maternal and child health in developing countries. The DHS provides the most reliable data source on child mortality across developing countries in terms of coverage, comparability, and data quality using extensive interviewer training, standardized measurement instruments and techniques, and instrument pretesting to ensure standardization and comparability across space and time (ICF Macro 2009). In order to realize the datasets, the DHS uses a two-stage stratified design: in the first stage, a number of clusters are selected with a probability proportional to the size from a frame list of enumeration areas (EAs) created from the most recent population census and in the second stage, a fixed number of households are selected from the complete list of households in each of the selected EAs. The households in a survey area are stratified according to type of residence (urban/rural) crossed by administrative/geographical regions (Aliaga and Ren 2006). All household members in a certain age group (usually women of reproductive age 15–49 and men age 15–59) in the selected households are interviewed. In this paper, the household members of interest were the mother, for which a complete birth and death history of her children was collected (including children’s birth date and death age, when applicable) and the children under age 5 born in the 5-year period before the survey.

The DHS–MIS was developed by the Monitoring and Evaluation Working Group of Roll Back Malaria, an international partnership coordinating global efforts to fight malaria, and collects national and regional or provincial data from a representative sample of respondents.

**Women continuing education after age 18 or 19**

Using the DHS household data, we calculated the percentage of women who continue their studies after age 18 or 19 as the ratio between the total number of *de jure* female members aged 18 or 19 or older who were attending school during the school year at the time of the survey and the total number of *de jure* female members between 18 or 19 years old and 24 years old. In Malawi, 12.1 %, 10.4 %, and 14.0 % of women continued their studies after 18 years old in 2000, 2004, and 2010, respectively; in Uganda, 10.7 %, 9.5 %, and 10.5 % of women continued their studies after 19 years old in 2000–2001, 2006, and 2011, respectively (we could not calculate the percentage for the 2009 survey due to limitations of the DHS–MIS data).

**Evidence of the differences in educational attainment**

Figure A2 shows the reverse cumulative distribution functions of years of education by mother’s year of birth for the treated and untreated women and the difference in these functions. In both countries, it is evident that the change in the distribution was greater among exposed women than among non-exposed women and that the former had more education compared with the latter.

**Indicators for the health knowledge pathway**

The first indicator, knowledge about contracting AIDS, was based on three questions on whether using condoms and having one sex partner can reduce the chances of contracting AIDS and whether a healthy-looking person can have AIDS. The second indicator, knowledge about transmission of AIDS, used two questions on whether a person can get AIDS by being bitten by a mosquito and by sharing food with a person who has it. The two items were coded 1 if the person cannot get AIDS under these circumstances and 0 otherwise. The third indicator, knowledge about ovulation, was based on one question on the timing of the ovulatory cycle and was coded 1 = correct answer “middle of cycle”, 0.5 = “after period ends” or “before period begins”, and 0 = incorrect.

**Table A1** Reduced form OLS estimates: Impact of UPE on 10 pathway indicators (one model per indicator per country)

|  |  |  | Malawi | | Uganda | |
| --- | --- | --- | --- | --- | --- | --- |
| Pathway indicator | |  | Young | Young x Program Intensity | Young | Young x Program Intensity |
| Socioeconomic Status | | |  |  |  |  |
|  | Wealth index | | 0.033 | -0.0004^†^ | 0.210^***^ | -0.0004^***^ |
|  |  |  | (0.023) | (0.0003) | (0.048) | (0.00001) |
|  | Medical care: money | | 0.059^*^ | 0.0001 | 0.117^**^ | -0.0002^*^ |
|  |  |  | (0.024) | (0.0004) | (0.040) | (0.0001) |
| Attitudes Toward Modern Health Services | | | |  |  |  |
|  | Use of modern contraception | | 0.081^***^ | -0.001^†^ | 0.022 | -0.0003^***^ |
|  |  |  | (0.021) | (0.0003) | (0.025) | (0.0001) |
| Personal Illness Control | | |  |  |  |  |
|  | Personal illness control | | -0.052 | 0.108^†^ | 0.067 | -0.058^***^ |
|  |  |  | (0.034) | (0.056) | (0.068) | (0.010) |
| Environmental Characteristics | | |  |  |  |  |
|  | Close to health facility | | 0.092^***^ | -0.001^*^ | 0.024 | 0.0001 |
|  |  |  | (0.022) | (0.0003) | (0.038) | (0.0001) |
| Health Knowledge | |  |  |  |  |  |
|  | Knowledge about getting AIDS | | 0.009 | 0.0001 | 0.075 | -0.0001 |
|  |  |  | (0.023) | (0.001) | (0.059) | (0.0001) |
|  | Knowledge about transmission of AIDS | | 0.070^**^ | -0.001 | -0.013 | -0.0001 |
|  |  |  | (0.026) | (0.0005) | (0.046) | (0.0001) |
|  | Knowledge about ovulation |  | -0.044^***^ | 0.0003 | -0.026 | -0.0001^†^ |
|  |  |  | (0.013) | (0.0003) | (0.027) | (0.0001) |
| Empowerment | |  |  |  |  |  |
|  | Decision-making | | -0.005 | 0.0002 | -0.146^*^ | -0.0002^†^ |
|  |  |  | (0.038) | (0.001) | (0.068) | (0.0001) |
|  | Empowered domestic violence | | 0.142^***^ | 0.001^**^ | 0.301^***^ | -0.0003^*^ |
|  |  |  | (0.030) | (0.001) | (0.055) | (0.0002) |
| *Notes*: The standard errors, shown in parentheses, are clustered at the district level. All regressions for mothers include religion (Malawi only), district fixed effects, interaction between the dummy variable for young and the district-specific number of girls in primary school before UPE, and interaction between the dummy variable for young and the number of primary school–aged children before UPE. All regressions for children further include child sex, birth order, and child year of birth fixed effects. Sample excludes missing information and Uganda DHS–MIS 2009 since these questions are not included in the DHS–MIS questionnaire (DHS restriction). The variable program intensity is expressed as percentage.  ^†^*p* < .10; **p* < .05; ***p* < .01; ****p* < .001 | | | | | | |

**References**

Aliaga, A., & Ren, R. (2006). *Optimal sample sizes for two-stage cluster sampling in Demographic and Health Surveys* (DHS Working Paper Series No. 30). Calverton, Maryland: ORC Macro.

Breglia, M. G., Guarcello, L., Rosati, F. C., & Ssennono, V. F. (2008). *Understanding children’s work in Uganda* (Working Paper No. 1). Washington, DC: World Bank.

Chimombo, J. G. C., Chibwanna, M., Dzimadzi, C., Kadzamira, E., Kunkwenzu, E., Kunje, D., & Namphota, D. (2000). *Classroom, school and home factors that negatively affect girls education in Malawi*. New York, NY: UNICEF.

Education Management Information Systems (EMIS). (2004). *Education statistics 2004*. Lilongwe, Malawi: Ministry of Education.

Education Management Information Systems (EMIS). (2006). *Education statistics 2006*. Lilongwe, Malawi: Department of Education Planning, Ministry of Education and Vocational Training.

Grogan, L. (2006). *Who benefits from universal primary education in Uganda?* (Unpublished paper). Guelph, Ontario: Department of Economics, University of Guelph. Retrieved from https://www.researchgate.net/publication/228571445_Who_benefits_from_universal_primary_education_in_Uganda

Grogan, L. (2009). Universal primary education and school entry in Uganda. *Journal of African Economies*, *18*(2), 183–211.

ICF Macro. (2009). *Demographic and Health Survey Interviewer’s Manual* (MEASURE DHS Basic Documentation No. 2). Calverton, Maryland: ICF Macro.

Keats, A. (2016). *Women’s schooling, fertility, and child health outcomes: Evidence from Uganda’s free primary education program* (Unpublished paper). Middletown, CT: Department of Economics, Wesleyan University.

Nakabugo, M. G., Byamugisha, A., & Bithaghalire, J. (2008). Future schooling in Uganda. *Journal of International Cooperation in Education*, *11*, 55–69.

Nambalirwa, S. (2010). *The implementation of universal primary education in Uganda* (Unpublished dissertation for Magister Administrationis). Faculty of Economic and Management Sciences, University of Pretoria, Pretoria, South Africa.

Tamusuza, A. (2011). Leaving school early: The quest for universal primary education in Uganda. *African Statistical Journal*, *13*, 110–151.

**Fig. A1** Fraction of girls in primary school by age in 1992 (Malawi) and 1995 (Uganda)


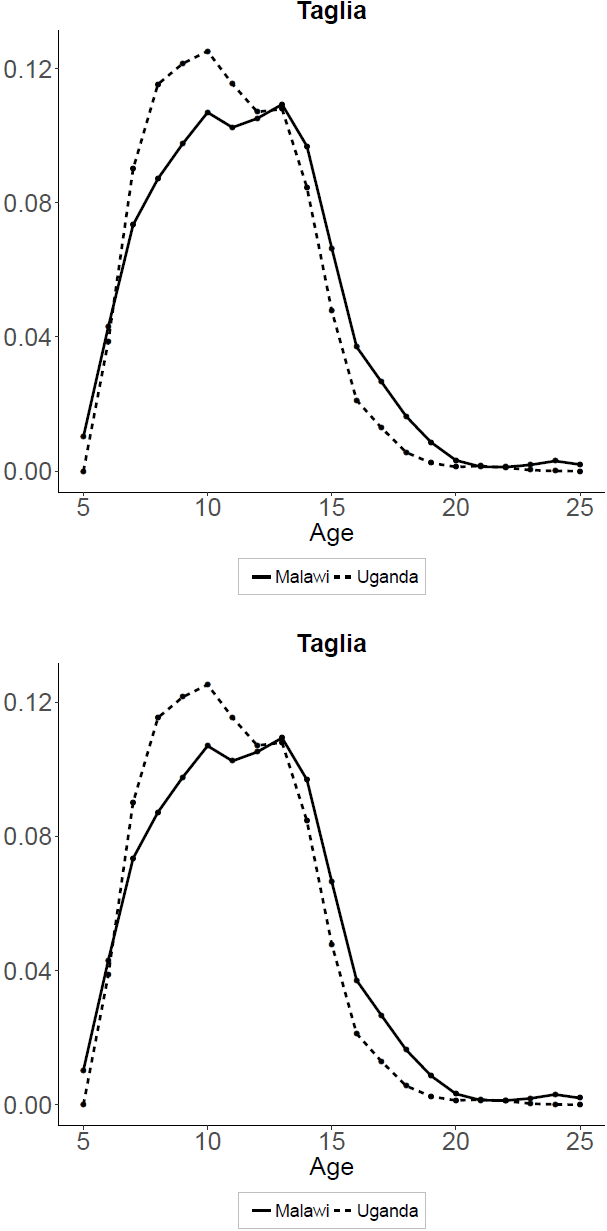


**Fig. A2** Reverse empirical cumulative distribution functions of education by mother’s year of birth


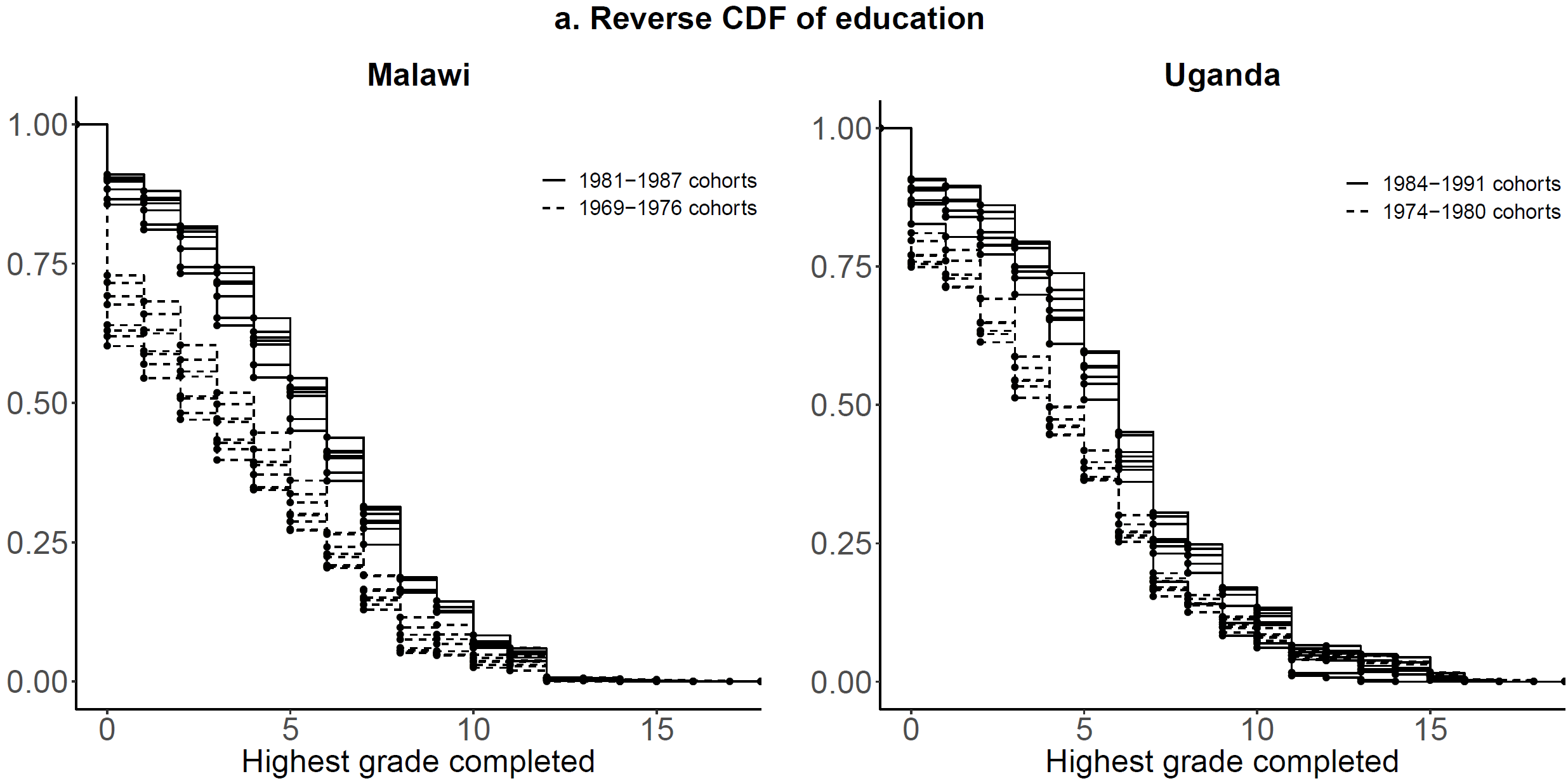


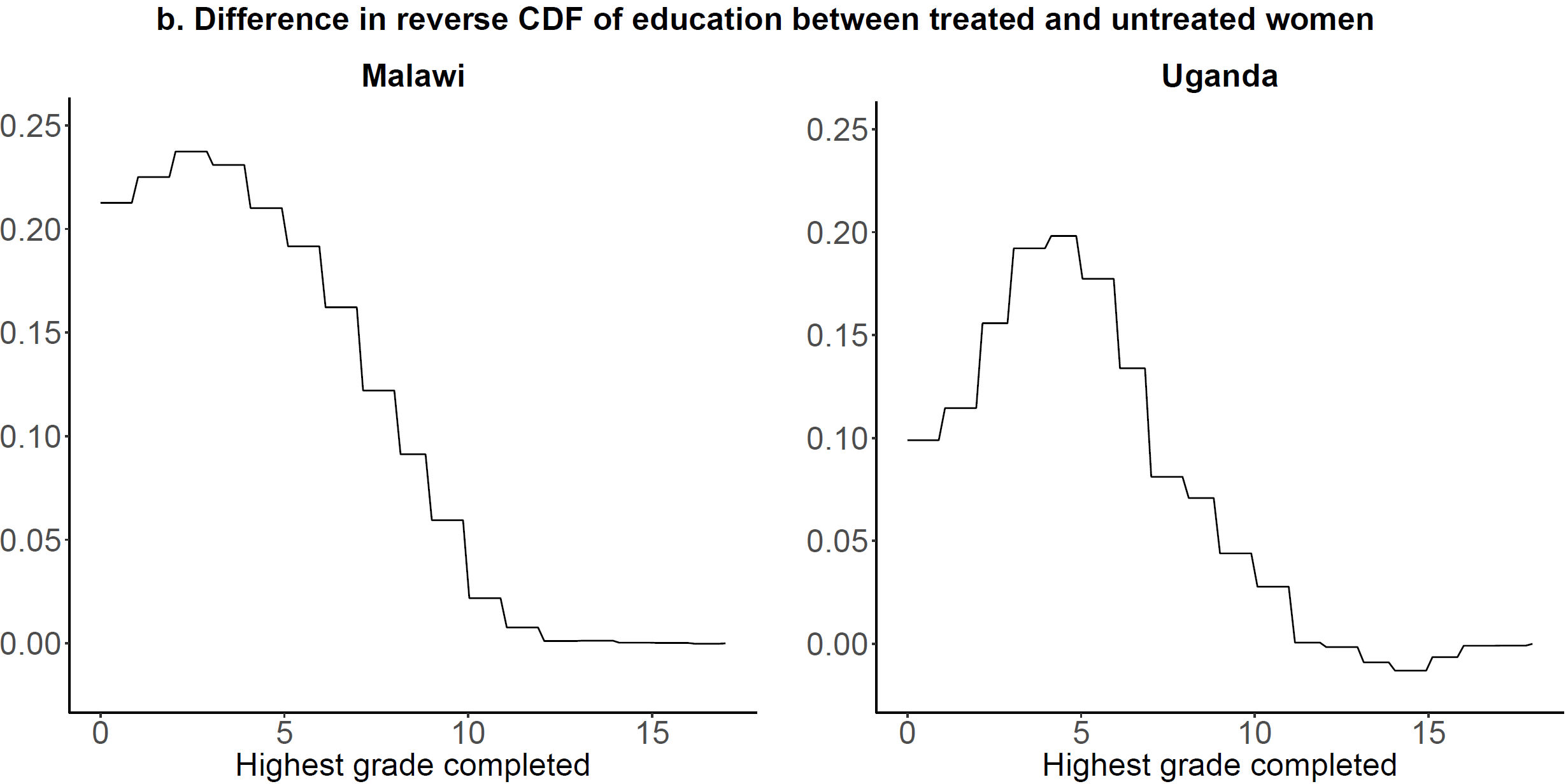


1. Clusters in the DHS we selected were geo-referenced, that is their coordinates were collected during the survey sample listing process using GPS receivers. [↑](#footnote-ref-1)
